# Subacute cognitive impairment after first-ever transient ischemic attack or ischemic stroke in young adults: The ODYSSEY study

**DOI:** 10.1177/23969873221132032

**Published:** 2022-10-31

**Authors:** Mijntje MI Schellekens, Esther M Boot, Jamie I Verhoeven, Merel S Ekker, Mayte E van Alebeek, Paul JAM Brouwers, Renate M Arntz, Gert W van Dijk, Rob AR Gons, Inge WM van Uden, Tom den Heijer, Paul LM de Kort, Karlijn F de Laat, Anouk van Norden, Sarah E Vermeer, Marian SG van Zagten, Robert J van Oostenbrugge, Marieke JH Wermer, Paul J Nederkoorn, Frank G van Rooij, Ido R van den Wijngaard, Frank-Erik de Leeuw, Roy PC Kessels, Anil M Tuladhar

**Affiliations:** 1 Department of Neurology, Radboud University Medical Centre, Donders Institute for Brain, Cognition and Behaviour, Nijmegen, The Netherlands; 2 Department of Neurology, Amphia Hospital, Breda, The Netherlands; 3 Department of Neurology, Medisch Spectrum Twente, Enschede, The Netherlands; 4 Department of Neurology, Canisius-Wilhelmina Hospital, Nijmegen, The Netherlands; 5 Department of Neurology, Catharina Hospital, Eindhoven, The Netherlands; 6 Department of Neurology, Franciscus Gasthuis & Vlietland, BA Rotterdam, The Netherlands; 7 Department of Neurology, Elisabeth-TweeSteden Hospital, Tilburg, Netherlands; 8 Department of Neurology, Haga Hospital, Den Haag, Netherlands; 9 Department of Neurology, Rijnstate Hospital, Arnhem, The Netherlands; 10 Department of Neurology, Jeroen Bosch Hospital, ‘s-Hertogenbosch, The Netherlands; 11 Department of Neurology, School for Mental Health and Neuroscience (MHeNs), Maastricht University Medical Centre, Maastricht, The Netherlands; 12 Department of Neurology, Leiden University Medical Centre, Leiden, The Netherlands; 13 Department of Neurology, Amsterdam University Medical Centre, location AMC, Amsterdam, The Netherlands; 14 Department of Neurology, Medical Centre Leeuwarden, Leeuwarden, The Netherlands; 15 Department of Neurology, Haaglanden Medical Center, The Hague, The Netherlands; 16 Radboud University, Donders Institute for Brain, Cognition and Behaviour, Center for Cognition, Nijmegen, The Netherlands; 17 Vincent van Gogh Institute for Psychiatry, Venray, The Netherlands

**Keywords:** Cognitive impairment, stroke in young adults, neuropsychological tests, subjective cognitive complaints

## Abstract

**Introduction:**

We aimed to investigate the prevalence of cognitive impairment in the subacute phase after transient ischemic attack (TIA) and ischemic stroke (IS), factors associated with a vascular cognitive disorder, and the prevalence of subjective cognitive complaints and their relation with objective cognitive performance.

**Patients and methods:**

In this multicenter prospective cohort study, we recruited patients with first-ever TIA and IS, aged 18–49 years, between 2013 and 2021 for cognitive assessment up to 6 months after index event. We calculated composite Z-scores for seven cognitive domains. We defined cognitive impairment as a composite Z-score < −1.5. We defined major vascular cognitive disorder as a Z-score < −2.0 in one or more cognitive domains.

**Results:**

Fifty three TIA and 545 IS patients completed cognitive assessment with mean time to assessment of 89.7 (SD 40.7) days. The median NIHSS at admission was 3 (interquartile range, 1–5). Cognitive impairment was common in five domains (up to 37%), with similar proportion in TIA and IS patients. Patients with major vascular cognitive disorder had a lower education level, higher NIHSS scores and more frequent lesions in the left frontotemporal lobe than without vascular cognitive disorder (*p* < 0.05 FDR-corrected). Subjective memory and executive cognitive complaints were present in about two-thirds of the patients, but were weakly associated with objective cognitive performance (β: −0.32 and −0.21, respectively).

**Discussion and conclusion:**

In the subacute phase after TIA or stroke in young adults, cognitive impairment and subjective cognitive complaints are prevalent, but they are weakly associated with each other.

## Introduction

Stroke in young adults affects at least 1.5 million people worldwide each year, with increasing incidence of stroke globally.^1,2^ Many young patients with a stroke experience lifelong disabling consequences, including cognitive impairment.^3^ Post-stroke cognitive impairment (PSCI) is an important clinical outcome in young patients as it affects their social life, quality of life and return to work, independent of physical recovery.^4^ However, data on PSCI in young patients are scarce.

Few short term (acute phase up to 12 months)^5–7^ and long term (up to several years)^3,8^ studies showed worse cognitive performance in patients with young stroke compared to healthy controls on a wide range of cognitive domains. Even in one-third of patients after a transient ischemic attack (TIA) aged 45–65 years, impairment in one or more cognitive domains was present within 3 months after their TIA.^9^ However, earlier studies in the subacute phase had a small sample size,^5–7^ did not cover all cognitive domains,^7^ included events with and without radiological evidence,^6,9^ or only included infratentorial infarcts.^6^ In addition, subjective cognitive complaints are prevalent in young adults measured in a small group of patients in the subacute phase and up to years after stroke, but its relation with objective cognitive impairment is uncertain.^10–12^

The goal of the present study therefore was (1) to investigate the cognitive performance prospectively covering all cognitive domains in the subacute phase (till 6 months) after a first-ever ischemic stroke (IS) or TIA with radiological evidence in a large cohort of young patients, (2) to explore clinical and radiological factors potentially associated with a cognitive disorder, and (3) to assess the prevalence of subjective cognitive complaints in the subacute phase and whether they predict objective cognitive performance.

## Patients and methods

### Patients and study design

This study is part of the “*O*bservational *D*utch *Y*oung *S*ymptomatic *S*trok*E* stud*Y”* (ODYSSEY), a multicenter prospective cohort study on the risk factors and prognosis of patients with a stroke in young adults.^13^ The present study comprises patients with first-ever TIA or IS with radiological evidence of cerebral ischemia, aged 18–49 years, included between May 2013 and February 2021. We defined acute stroke as a rapidly evolving focal neurological deficit, without positive phenomena, with a vascular cause, lasting for more than 24 h. We defined TIA similarly with a duration of clinical symptoms less than 24 h with radiological evidence of cerebral ischemia (tissue-based definition). Exclusion criteria were a history of stroke, TIA, retinal infarction, and cerebral venous sinus thrombosis. Detailed information on the data collection is described elsewhere.^13^ The Medical Review Ethics Committee region Arnhem-Nijmegen approved the study. We obtained written informed consent from all participants. If the patient was unable to provide informed consent, consent was provided by the patient’s legally acceptable representative.

### Cognitive assessment

Neuropsychological tests were administrated up to 6 months after the index event. The cognitive assessment included tests used in other large scale epidemiologic studies covering the most relevant cognitive domains.^14,15^ The following cognitive domains were examined: *Episodic memory* (3-trial version of the Rey Auditory Verbal Learning Test), *Processing speed* (the written version of the Symbol-Digit Modalities Test, the abbreviated Stroop Color Word Test, parts I and II), *Visuoconstruction* (Rey-Osterrieth Complex Figure (ROCF)-copy trial), *Executive functioning* (Fluency test, Stroop interference score, Brixton Spatial Anticipation Test), *Visual neglect* (Star Cancelation of the Behavioral Inattention Test), *Language deficits* (Short Token Test), *Attention and working memory* (Digit Span subtest from the Wechsler adult Intelligence Scale – Fourth Edition). Global cognitive functioning was examined with the Mini Mental State Examination. If a specific test was not performed due to technical problems, physical disability, cognitive impairment that prevented the patient from understanding the instruction, or refusal of the patient, only the reliable and valid administered tests were included. Supplemental Table 2 provides the reasons for non-completion for each test. We calculated composite scores to account for speed-accuracy trade-off on the Stroop test (accuracy(%)/reaction time). We computed Stroop interference by dividing the composite Stroop part III score by the mean of the composite scores of parts I and II. To prevent potential bias in scoring the ROCF, two researchers independently rated 10% of the complex figures, with high inter-rater reliability using the Pearson’s correlation coefficients (r_s_ = 0.95).^16^

For most tests, we used the normative data from the Advanced Neuropsychological Diagnostics Infrastructure (ANDI) that includes data from up to 26,000 healthy individuals from all ages, enabling fine-grained adjustment for age, sex and/or education level, where appropriate.^17^ For the written version of the Symbol-Digit Modalities Test, we used the normative data from the test manual (n = 1307),^18^ adjusted for age and education level. For the abbreviated Stroop Color Word Test, we used age- and education-matched control data from our earlier young-stroke study (n = 146).^3^ We used healthy controls from another stroke study for the Star Cancelation test (n = 63).^19^

We converted raw test scores to Z-scores per test for each participant based on the normative data from the ANDI dataset, or based on the mean and the SD of control data, adjusted for age and education level (age- and education-adjusted normative mean: *Z* = 0; SD = 1). In four patients, education level was missing, we used simple imputation with the median (education category 5, i.e. middle school/secondary vocational training) for these missing values. We corrected Z-scores >3 or <−3 to 3 and −3 respectively, to correct for outliers. Next, averaging Z-scores of cognitive tests that reflected the same cognitive domain resulted in a composite Z-score per cognitive domain. If one test of a particular domain was missing, the domain score was based on the remaining tests of that domain. We defined cognitive impairment on a test as a Z-score of <−1.5 (i.e. reflecting a performance level of more than 1.5 SD below the age- and education-adjusted normative mean) on that particular test. We defined cognitive impairment on a domain as a composite Z-score of <−1.5 and a below average performance as a composite Z-score between −1.0 and −1.5.^20^

To compare patients on various clinical and radiological parameters, we used the diagnostic criteria for vascular cognitive disorder (VCD) of the International Society for Vascular Behavioral and Cognitive Disorders (VASCOG). We defined mild VCD as a composite Z-score of between −1.5 and −2.0 in one or more cognitive domains (representing 4.4% of the normal population).^21^ We defined major VCD as a composite Z-score of <−2.0, in one more cognitive domains (representing 2.3% of the normal population). These criteria are more conservative and have a higher specificity than the VASCOG cut-off criteria, which define mild VCD as a Z-score in one or more cognitive domains between −1.0 and −2.0.^22^ This interval, however, represents 13.6% of the normal population, resulting in a poor specificity and the risk of a too high proportion of false positive diagnoses.

### Subjective cognitive assessment

We used a 15-item semi-structured interview on subjective cognitive complaints, which has been applied in previous research,^11^ to assess the presence of subjective cognitive complaints in the past month. Subjective cognitive complaints were considered present when a participant scored a “2” (moderate) or higher on a scale of 0–3 on at least 1 item or scored a “1” (present) on 1 item with dichotomous answers. Next, we calculated the total scores of subjective memory complaints (two questions with a 4-point scale and 8 questions witch dichotomous answers) and subjective executive complaints (three questions with a 4-point scale).

### Other measurements

We scored level of education with a Dutch scoring system, using seven categories (1 = less than primary school; 7 = university degree),^23^ comparable with the UNESCO international classification of education levels.^24^ Symptoms of depression and fatigue were assessed using the Mini International Neuropsychiatric Interview (MINI),^25^ and the subscale Subjective Fatigue of the revises Checklist on Individual Strength (CIS-20R).^26^

We evaluated functional outcome at the time of the cognitive assessment using the Barthel Index^27^ and modified Rankins Scale (mRS).^28^ We defined good functional outcome as a mRS score of 0–1 and a Barthel Index of ⩾85.

Furthermore, we assessed the etiology of stroke (based on modified Trial of ORG 10172 in Acute Stroke Treatment; TOAST)^29^ and severity at discharge (National Institutes of Health Stroke Scale; NIHSS)^30^ retrospectively using a validated approach,^31,32^ because this scale was not used in all medical files.

Lesion locations and vascular territories were visually scored on the available imaging modalities.

We determined whether there was recurrent stroke before the cognitive assessment based on patient records or a telephone interview.

## Statistical analyses

We performed all statistical analyses with RStudio 3.6.2 and IBM SPSS Statistics 25. We compared baseline characteristics of patients with cognitive assessment and patients without cognitive assessment using a Pearson’s χ^2^ test, Mann-Whitney *U* test, or Student’s *t-*test when appropriate.

We used one-sample *t*-test with one-tailed *p*-values for the composite Z-scores of each cognitive domain in TIA and IS patients separately to determine if they were lower than the age, education and/or sex-adjusted control. We investigated differences in composite Z-scores between TIA and IS with Student’s *t*-test. We used a one-way analysis of covariance (ANCOVA) to determine whether there were significant differences in composite Z-scores between each stroke subtype adjusting for fatigue severity and symptoms of depression.

We used a Pearson’s χ^2^ test (or Fisher’s exact test when an expected cell count was less than 5) to investigate differences in TIA and IS patients in the proportion of participants with a below average performance or cognitive impairment.

We compared patients without VCD, with mild VCD and major VCD using a one-way analysis of variance (ANOVA), Pearson’s χ^2^ test or Kruskal-Wallis test when appropriate. If there was a significant difference between the groups, we used the Pearson’s χ^2^ test or the Mann-Whitney *U* test to perform pairwise comparisons as post-hoc analysis. Furthermore, we compared patients without VCD, with mild VCD and major VCD on infarcts involving the left frontotemporal lobe, left thalamus, or right parietal lobe, since lesions in these locations are strongly associated with post-stroke cognitive impairment.^33^

We determined the prevalence of subjective memory complaints and subjective executive complaints. Subsequently, the distribution of the total scores of subjective memory and executive complaints is reported as a measure of severity of subjective complaints. The association between subjective cognitive complaints and objective performance was calculated in two ways. First, we determined the association between total scores of subjective memory complaints and the domain score of attention and working memory, and between subjective executive failure and the domain score of executive functioning using linear regression, while adjusting for depression and fatigue. Next, we determined the association between the total scores of subjective cognitive complaints and the number of cognitively impaired tasks, using linear regression.

To explore the effect of differences in time from index event to the neuropsychological assessment on the composite Z-score in each domain we used Pearson correlation analysis. Next, we performed a median split in “time from index event to cognitive assessment” and compared the groups with (1) the composite Z-scores in each domain using a Student’s t-test and (2) the proportion of patients with cognitive impairment on a domain using Pearson’s χ^2^ test.

To correct for multiple comparisons, we applied a Benjamini-Hochberg procedure, with false discovery rate Q set at 0.05.^34^ We reported two-tailed *p*-values, unless the use of a one-tailed *p*-value is specifically stated.

To investigate whether a recurrent event influenced the results, we performed post-hoc analyses in which we conducted all above described analyses after excluding patients with a recurrent stroke before the first cognitive assessment.

## Results

**Figure 1.**
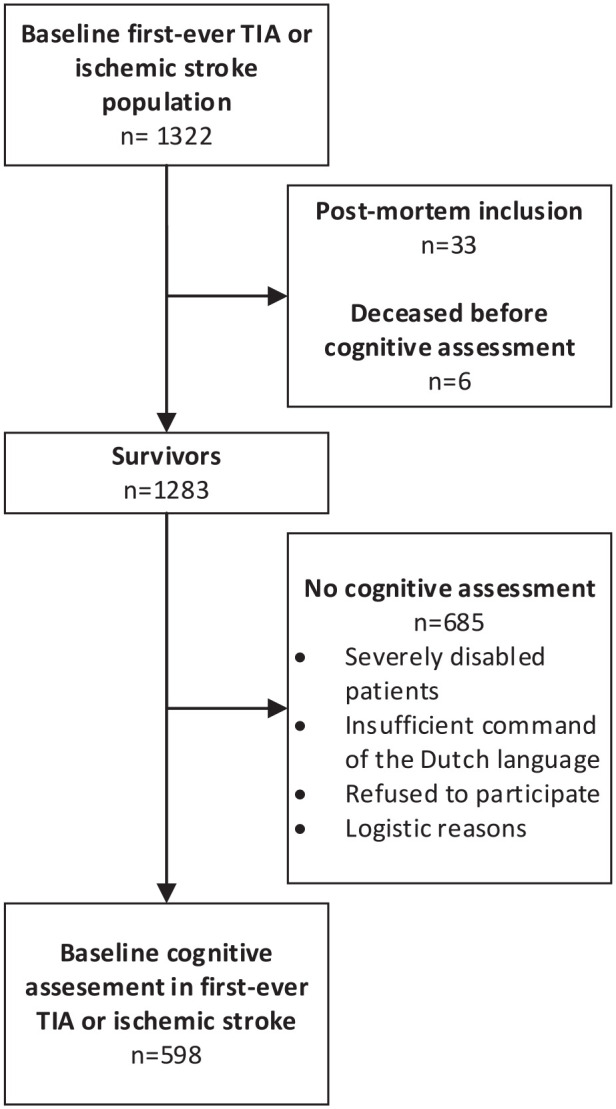
Flowchart of the study population.

This study consisted of 53 TIA and 545 IS patients (Figure 1). Baseline characteristics of the study population are described in Table 1 and neuropsychological test scores are presented in Table 2, both stratified by the type of event. Mean age of patients at stroke onset was 41.7 (SD 7.7) years, 48.2% were women, and the median NIHSS at admission was 3 (interquartile range, 1–5). Mean time from index event to cognitive assessment was 89.7 (SD 40.7) days. Baseline characteristics of patients with (*n* = 598) or without cognitive assessment (*n* = 685) are presented in Supplemental table 1.

**Table 1.** Baseline characteristics.

	All participants (*n* = 598)	TIA (*n* = 53)	Ischemic stroke (*n* = 545)
Mean age at index event, years (SD)	41.7 (7.7)	39.3 (9.1)	41.9 (7.5)
Men, N (%)	310 (51.8)	25 (47.2)	285 (52.3)
Mean time to assessment, days (SD)	89.7 (40.7)	92.3 (43.4)	89.5 (40.4)
Lesion location, right/left, N (%)			
Anterior	349 (58.4)	40 (75.5)	309 (56.7)
MCA	185(30.9)/149(24.9)	17(32.1)/22(41.5)	168(30.8)/127(23.3)
ACA	1 (0.2)/6 (1.0)	0 (0)/0 (0)	1 (0.2)/6 (1.1)
MCA and ACA	5 (0.8)/3(0.5)	1 (1.9)/0 (0)	4 (0.7)/3 (0.6)
Posterior	184 (30.8)	9 (17.0)	175 (32.1)
PCA	25 (4.2)/39 (6.5)	1 (1.9)/4 (7.5)	24 (4.4)/35 (6.4)
Vertebrobasilar	120 (20.1)	4 (7.5)	116 (21.3)
Watershed	3 (0.5)/4 (0.7)	0 (0)/ 0 (0)	3 (0.6)/4 (0.7)
Multiple	58 (9.7)	4 (7.5)	54 (9.9)
Median education level (IQR)	5 (5–6)	5 (5–6)	5 (5–6)
Median NIHSS score at admission (IQR)	3 (1–5)	0 (0–1)	3 (1–5)
Median NIHSS score at discharge (IQR)	1 (0–2)	0 (0–0)	1 (0–2)
Mean barthel Index at assessment (SD)	98.3 (7.0)	99.7 (1.2)	98.1 (7.4)
Good outcome (BI ⩾ 85), N (%)	550 (96.3)	51 (100)	499 (96.0)
Median mRS at assessment (IQR)	1 (1–2)	1 (0–1)	1 (1–2)
Good outcome (mRS 0–1), N (%)	377 (65.6)	43 (82.7)	334 (63.9)
Median MMSE (IQR)	28 (26–29)	28 (27–29)	28 (26–29)
MINI- symptoms of depression present, N (%)	54 (9.3)	3 (5.7)	51 (9.7)
Mean CIS-20R-fatigue severity (SD)	32.9 (11.9)	32.8 (12.8)	32.9 (11.8)
Mild fatigue 27–35, N (%)	135 (27.7)	12 (26.1)	123 (27.8)
Severe fatigue ⩾36, N (%)	205 (42.0)	19 (41.3)	186 (42.1)
TOAST, N (%)			
Atherothrombotic	25 (4.2)	0 (0.0)	25 (4.6)
Likely atherothrombotic	70 (11.7)	4 (7.5)	66 (12.1)
Small vessel	85 (14.2)	3 (5.7)	82 (15.0)
Cardioembolic	97 (16.2)	17 (32.1)	78 (14.7)
Rare causes	125 (20.9)	9 (17.0)	116 (21.3)
Multiple causes	37 (6.2)	4 (7.5)	33 (6.1)
Cryptogenic	159 (26.6)	16 (30.2)	143 (26.2)

IQR: interquartile range; MCA: Middle Cerebral Artery; ACA: Anterior Cerebral Artery; PCA: Posterior Cerebral Artery; NIHSS: National Institutes of Health Stroke Scale; mRS: modified Rankin Scale; MMSE: Mini-Mental State Examination; MINI: Mini International Neuropsychiatric Interview; CIS-20R: Checklist Individual Strength; TOAST: Trial of ORG 10172 in Acute Stroke Treatment.

Education category 5, i.e. middle school/secondary vocational training.

Missing data: NIHSS at admission 3 (0.5%); NIHSS at discharge 3 (0.5%); Barthel index 27 (4.5%); mRS 23 (3.8%); MMSE 32 (5.4%); MINI – symptoms of depression 20 (3.3%); CIS-20R-fatigue 110 (18.4%).

**Table 2.** Raw neuropsychological test scores and percentage of patients with cognitive impairment on a test.

Cognitive domain & test	Participants (*n* = 598)	Percent cognitive impaired^a^	TIA (*n* = 53)	Percent cognitive impaired^a^	Ischemic stroke (*n* = 545)	Percent cognitive impaired^a^
Episodic memory						
RAVLT trial 1–3	21.3 (6.2)	25.0	21.3 (4.5)	28.3	21.3 (6.3)	24.6
RAVLT delayed recall	6.7 (2.9)	20.6	6.7 (2.6)	10	6.7 (3.0)	20.7
Processing speed						
SDMT	49.9 (12.0)	27.3	55.0 (9.9)	15.4	49.4 (12.1)	28.5
Stroop part I^b^	4.2 (1.0)	23.1	4.5 (0.9)	7.8	4.1 (1.0)	24.6
Stroop part II^b^	3.3 (0.8)	23.4	3.6 (0.7)	9.8	3.3 (0.8)	24.7
Visuoconstruction						
ROCF copy	29.9 (4.7)	37.0	31.7 (3.7)	28.3	29.7 (4.8)	37.9
Executive functioning						
Verbal fluency	19.4 (5.0)	13.5	20.6 (4.4)	9.4	19.2 (5.0)	13.9
Stroop interference^b^	0.57 (0.1)	7.0	0.59 (0.1)	0.0	0.57 (0.1)	40.0
Brixton test	12.7 (6.1)	8.4	11.0 (4.7)	1.9	12.9 (6.2)	9.1
Visual neglect						
Star cancelation	53.6 (1.3)	6.8	53.9 (0.3)	0.0	53.6 (1.4)	7.5
Language deficits						
Short token test	19.5 (1.9)	18.7	19.6 (2.2)	17.0	19.5 (1.9)	18.8
Attention and working memory						
Digit span test	24.6 (5.1)	22.8	25.3 (4.8)	23.1	24.5 (5.1)	22.8

RAVLT: Rey Auditory Verbal Learning Test; SDMT: Symbol-Digit Modalities Test; ROCF: Rey-Osterrieth Complex Figure.

Data were expressed as mean (SD).

Test not valid/performed: RAVLT trial 1–3: *n* = 9 (1.5%); RAVLT delayed recall *n* = 19 (3.2%); SDMT *n* = 37 (6.2%); Stroop part I *n* = 26 (4.3%); Stroop part II *n* = 25 (4.2%); ROCF copy *n* = 31 (5.2%); Verbal fluency *n* = 13 (2.2%); Stroop interference *n* = 29 (4.8%); Brixton test *n* = 17 (2.8%); Star cancelation *n* = 14 (2.3%); Short token test *n* = 30 (5.0%); Digit span test *n* = 28 (4.7%).

a Percent cognitive impaired: the percentage of the patients with a Z-score of <−1.5 on the test.

b Speed-accuracy composite score. Higher scores indicate better performance on all measures, except for the Brixton test (number of errors).

### Cognitive outcome after TIA or ischemic stroke

**Figure 2.**
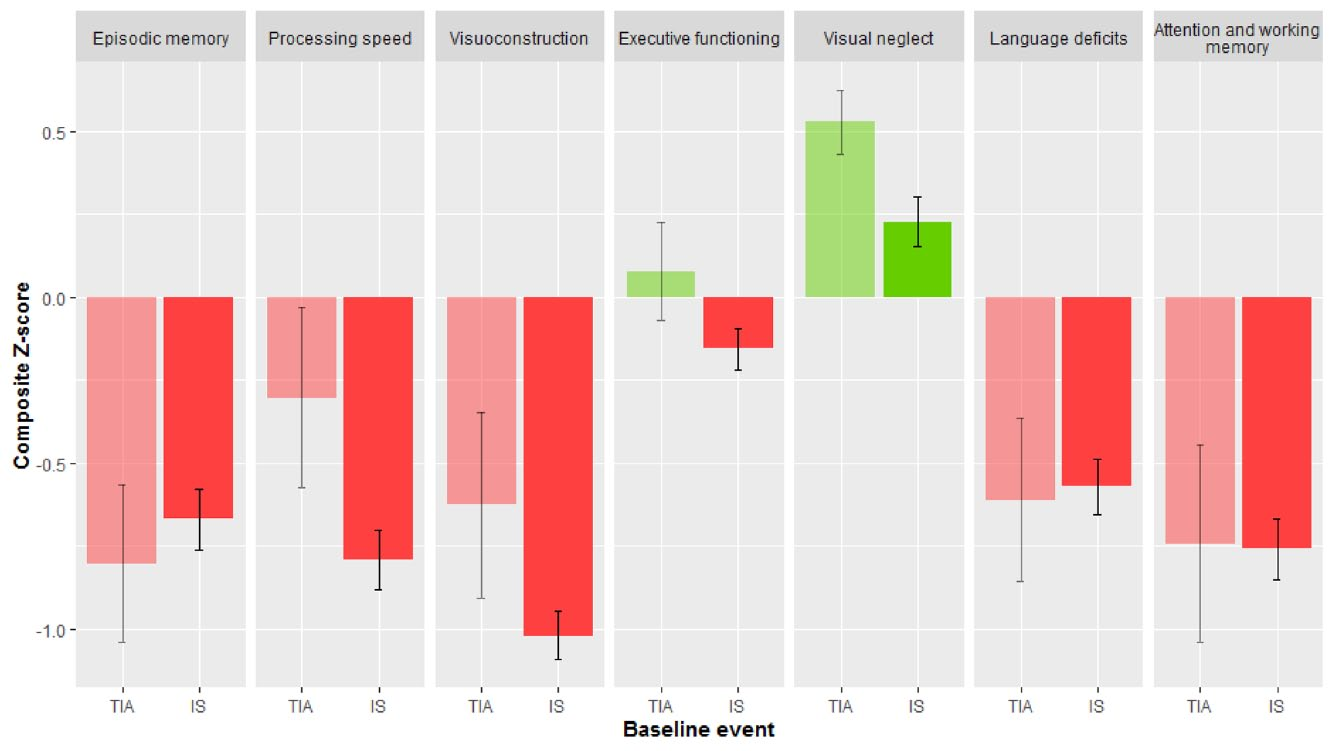
Cognitive performance after first-ever TIA or ischemic stroke. Cognitive performance after TIA or ischemic stroke in young adults. Mean composite Z-score (95% confidence interval) per cognitive domain. Z-scores were based on the raw scores of our patients compared with a control group or normative data. IS: ischemic stroke. Missing values in different domains: 0.2%−5.7%.

TIA and IS patients had a mean composite Z-score below the normative mean on respectively 5 and 6 domains (Figure 2). IS patients had worse cognitive performance than TIA patients on processing speed (*p* = 0.001), visuoconstruction (*p* = 0.002), executive functioning (*p* = 0.004), and visual neglect (*p* = 0.02). After additional adjustment for fatigue severity and symptoms of depression, IS patients had worse cognitive performance than TIA patients on processing speed (*p* = 0.008) and visuoconstruction (*p* = 0.007). Time from index event to neuropsychological assessment was not significantly associated with cognitive performance in any of the domains. In addition, there were no differences in composite Z-scores on any of the cognitive domains using a median split in “time from index event to cognitive assessment” (i.e. neuropsychological assessment within 83 days vs after 83 days).

### Below-average performance and cognitive impairment after TIA or ischemic stroke

**Figure 3.**
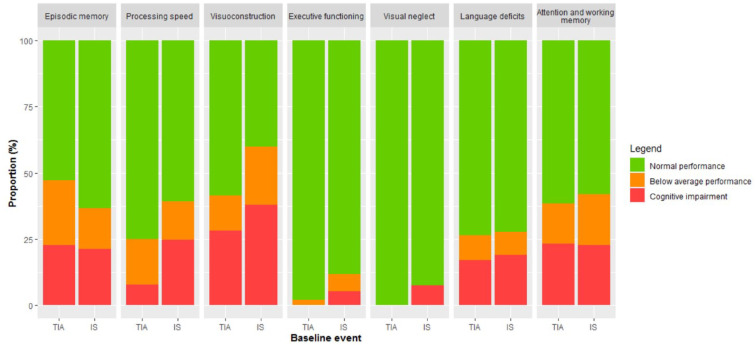
Below average performance and cognitive impairment stratified by event. The proportion of patients (%) with TIA and ischemic stroke at young age with below average performance (composite Z-score between −1.5 and −1.0) or a cognitive impairment (composite Z-score <−1.5). IS: ischemic stroke.

The percentage of patients with cognitive impairment on each test is presented in Table 2. The total number of cognitively impaired tests in a patient is described in Supplemental Table 3. Among TIA and IS patients, there was a high proportion of patients with below-average performance and cognitive impairment (Figure 3). Cognitive impairment in episodic memory (21.4%), processing speed (23.3%), visuoconstruction (37.0%), language deficits (18.7%), and attention and working memory (22.8%) were most common. There were no significant differences between TIA and IS patients in the proportion of patients with below-average performance or cognitive impairment. There were no significant differences between patients who completed the cognitive assessment within 83 days after the event versus more than 83 days after the event with respect to the proportion of patients with a cognitive impairment.

### Mild and major vascular cognitive disorder

30.2% of TIA patients had mild VCD and 28.3% had major VCD. In IS patients, the prevalence of mild and major VCD was 33.8% and 34.3% respectively. Differences in baseline and imaging characteristics between patients without VCD, mild VCD and major VCD are described in Table 3. Patients with major VCD had a lower education level, had more frequent lesions in the left frontotemporal lobe, a higher NIHSS score at admission and at discharge than patients without VCD (all *p* < 0.05 FDR-corrected).

**Table 3.** Patients with mild and major vascular cognitive disorder and no cognitive disorder.

Characteristics	Without VCD (*n* = 196)	Mild VCD (*n* = 200)	Major VCD (*n* = 202)
Mean age at index event, years (SD)	42.6 (6.9)	42.3 (7.0)	44.5 (8.8)
Mean time to assessment, days (SD)	93.4 (42.0)	89.6 (42.3)	86.3 (37.5)
Median education level (IQR)^a^	5 (5–6)	5 (5–6)	5 (5–6)
Ischemic stroke, N (%)	174 (88.8)	184 (92.0)	187 (92.6)
Territorial lesion, N (%)	144 (73.5)	150 (75.0)	151 (74.8)
Lesion location, N (%)			
Left frontotemporal lobe^b^	36 (18.4)	38 (19.0)	58 (28.7)
Left thalamus	18 (9.2)	20 (10.0)	15 (7.4)
Right parietal lobe	19 (9.7)	29 (14.5)	29 (14.4)
Median NIHSS score at admission (IQR)^c^	2 (1–5)	2 (1–5)	3 (1–6)
Median NIHSS score at discharge (IQR)^a^	0 (0–2)	1 (0–2)	1 (0–3)
Thrombolysis, N (%)	52 (26.6)	52 (26.1)	47 (23.2)
Thrombectomy, N (%)	13 (6.6)	9 (4.5)	16 (7.9)
BI good outcome (⩾85), N (%)	185 (96.9)	187 (97.9)	178 (94.2)
mRS good outcome (0–1), N (%)	135 (70.7)	128 (66.0)	114 (60.0)
MINI - symptoms of depression present, N (%)	10 (5.2)	23 (11.8)	21 (11.1)
Mean CIS-20R-fatigue severity, (SD)	31.4 (11.7)	33.1 (11.8)	33.5 (12.2)
Mild fatigue 27–35, N (%)	45 (26.5)	45 (27.4)	45 (29.2)
Severe fatigue ⩾36, N (%)	66 (38.8)	70 (42.7)	69 (44.8)
TOAST, N (%)			
Atherothrombotic	7 (3.6)	6 (3.0)	12 (5.9)
Likely atherothrombotic	21 (10.7)	26 (13.0)	23 (11.4)
Small vessel	25 (12.8)	28 (14.0)	32 (15.8)
Cardioembolic	38 (19.4)	31 (15.5)	28 (13.9)
Rare causes	41 (20.9)	36 (18.0)	48 (23.8)
Multiple causes	13 (6.6)	15 (7.5)	9 (4.5)
Cryptogenic	51 (26.0)	58 (29.0)	50 (24.8)

IQR: interquartile range. VCD: vascular cognitive disorder; NIHSS: National Institutes of Health Stroke Scale; BI: Barthel Index; mRS: modified Rankin Scale; MINI: Mini International Neuropsychiatric Interview; CIS-20R: Checklist Individual Strength.

Education category 5, i.e. middle school/secondary vocational training.

Missing data: NIHSS at admission three (0.5%); NIHSS at discharge 3 (0.5%); thrombolysis 2 (0.3%); thrombectomy 2 (0.3%); Barthel index 27 (4.5%); mRS 23 (3.8%); MINI- symptoms of depression 20 (3.3%); CIS-20R-fatigue 110 (18.4%).

a Indicating significant difference between without VCD and mild VCD, and without VCD and major VCD after Benjamini-Hochberg correction.

b Indicating significant difference between without VCD and major VCD, and mild VCD and major VCD after Benjamini-Hochberg correction.

c Indicating significant difference between without VCD and major VCD after Benjamini-Hochberg correction.

### Subjective cognitive complaints

Subjective memory complaints were present in 60.4% of the TIA patients and 71.5% of the IS patients. Subjective executive complaints were present in 45.3% of the TIA and 63.5% of the IS patients. Supplemental Figure 1 shows the distribution of total scores of subjective memory and executive complaints. Higher scores of subjective memory complaints were associated with lower cognitive performance on the cognitive domain attention and working memory in the overall study population (β: −0.32, *p* < 0.001) and for the IS patients (β: −0.34, *p* < 0.001), but not for TIA patients. However, the effect sizes were small with an *R*^2^_adjusted_ of 0.10 and 0.11, respectively. Higher scores of the subjective executive complaints were associated with lower cognitive performance on the cognitive domain executive functioning in the overall study population (β: −0.21, *p* < 0.001) and for the IS patients (β: −0.20, *p* < 0.001), but not for TIA patients. Again, the effect sizes were small with an *R*^2^_adjusted_ of 0.04 in both the analyses. Supplemental Figure 2 shows the association between the total scores of subjective cognitive complaints and the number of cognitively impaired tasks. The effect size of this relation is small, with an *R*^2^ of 0.06 (*p* < 0.05).

### Recurrent stroke

After excluding patients who had a recurrent TIA (*n* = 4) or IS (*n* = 24) before the cognitive assessment, we no longer found a significant difference in cognitive performance between TIA and IS on processing speed, after adjustment for fatigue severity and symptoms of depression. In addition, we found a lower proportion of patients with symptoms of depression in the group without VCD compared to the group with mild VCD (*p* = 0.015). Excluding patients with a recurrent stroke did not influence the other results.

## Discussion

In this large prospective study in young patients with TIA or IS, we found that (1) a high proportion of both TIA and IS patients showed worse cognitive performance on a wide range of cognitive domains in the subacute phase (up to 6 months after index event) compared to healthy controls, (2) higher NIHSS score at admission and discharge, stroke lesion in the left frontal lobe and lower education level were more common in patients with a major VCD than in patients without VCD, and (3) subjective cognitive complaints were prevalent, but were weakly associated with objective cognitive performance.

This study has multiple strengths. First, this is a multicenter prospective study with large sample size consisting of first-ever TIA and IS patients at a young age. Second, both TIA and IS were supported by radiological evidence, which reduces the risk of stroke mimics. Third, we used extensive neuropsychological testing (though a weakness is that this may prevent people from participating because of the extensiveness of the examination), as well as comprehensive questionnaires on mood and subjective cognitive complaints, with limited missing data. Finally, we used the normative data from the ANDI database, containing scores on neuropsychological tests from a large group of healthy controls.

However, several limitations need to be addressed. First, cognitive data of patients who were unable (for example severe aphasia) or refused to participate were lacking. This selection bias could affect the results, as patients without cognitive assessment had higher NIHSS scores at admission and discharge, but we expect that this bias, if any, would most likely lead to underestimation of the actual deficits. A shorter domain-specific assessment such as the Oxford Cognitive Screen,^35^ less confounded by aphasia, may be used in future research to get an estimate of the cognitive status of individuals unable to complete a full comprehensive neuropsychological assessment. Second, due to logistic reasons, not all the neuropsychological tests were performed at the exact same time point after stroke. Time between the stroke and neuropsychological assessment might affect the cognitive performance, as recovery may have occurred in some patients. Nevertheless, all cognitive tests were performed within 6 months after the event and time from index event to assessment was unrelated to the test performance nor was it different between without, mild, and major cognitive disorder. Third, premorbid cognitive performance of our patients is unknown. However, all patients are under the age of 50 and we expect that other neurodegenerative disorders will be negligible. Fourth, the lack of a strong association between subjective cognitive complaints and objective cognitive performance could have been due to the instrument used to measure it, as the semi-structured interview on subjective cognitive complaints is a generic instrument. A stroke-specific instrument, such as the Checklist of Cognitive an Emotional Consequences after stroke (CLCE-24), may be more preferable for future studies.^12^ Finally, our patients with stroke at young age scored well on the Star Cancelation test, leading to better performance on visual neglect compared to controls. For this test, we used a control group as reference, who were on average older than ours.^19^ However, the main advantage of using this control group is to obtain a continuous outcome measure.

We showed that cognitive impairment was common in young patients after IS and TIA with an equal proportion of patients with a below average performance and cognitive impairment. This suggest that TIA patients exhibit cognitive impairment similar to IS patients, even after full recovery from focal neurological symptoms. In our study, all TIA patients had recent cerebral ischemia on MRI. This could cause temporary or permanent disruption of the brain network, resulting in cognitive impairment. In addition, anxiety, depression, and fatigue may also contribute to cognitive impairment.

Consistent with other studies with similar severity of stroke in terms of NIHSS and mRS, deficits in memory, language deficits, attention, and especially processing speed were most common.^3,5,7–9^ Our patients performed relatively well on executive functioning. This domain is partly evaluated based on the performance of the Stroop parts I and II, which was scored relatively low in a high proportion of participants, resulting in relatively unimpaired Stroop interference scores. In addition, another explanation for this result is the use and the timing of different subtests and operationalization of the executive domain in studies,^3,7^ which may result in different outcomes. For instance, both our Stroop interference score (which adjusts for baseline processing speed by computing a ratio score) and the Brixton test (which is not timed and does not require a motor response) are not confounded by the patients’ processing speed deficits, making them a more process-pure measure of executive function.

The results of this study provide evidence for high prevalence of mild or major VCD in the subacute phase. Up to two-third of our patients had mild or major VCD, even after using strict criteria for VCD with a higher specificity. Factors that are related to major VCD, are lower education level, higher NIHSS scores at admission and at discharge and lesions in the left frontotemporal lobe. Previous studies have found that left hemisphere stroke is more frequent associated with cognitive impairment.^3^ This may be explained by the involvement of language area as the most cognitive tests are heavily language-based. Note that in this study, we only scored lesion locations visually. Lesion symptom mapping might be more specific to support this hypothesis. In addition, disrupted brain network involved in cognitive process due to strategic infarct locations and lower remote white matter integrity could lead to the development of PSCI.^33,36^ These findings suggest that if one of these factors is present, clinicians should be aware that PSCI might be present.

Subjective memory and executive complaints were present in about two-third of the participants. The high prevalence of subjective cognitive complaints may be due to the cut-off values (i.e. one positive answer already results in classification as “subjective cognitive failures present.” This makes it a very sensitive method for assessing subjective cognitive complaints, but not very specific.

The subjective cognitive complaints scores were only weakly associated with objective cognitive performance. This suggests that in addition to objective cognitive impairment other factors, including distress-related psychological factors, coping and personality traits,^37,38^ may also be linked to subjective cognitive complaints. Interventions such as psycho-education, physical therapy and cognitive rehabilitation and cognitive rehabilitation might be a valuable addition to stroke rehabilitation reducing subjective cognitive complaints.^37^ This highlights the importance of the evaluation of objective and subjective cognitive performance in the first few months after the stroke, since these information may helpful to provide adequate patient-centered stroke care.

## Conclusion

In conclusion, we showed that cognitive impairment on a wide range of cognitive domains and subjective cognitive complaints were prevalent in young patients after TIA and IS. However, they are only weakly associated with each other. Both neuropsychological assessment in the (sub)acute phase and subjective cognitive assessment may be considered in young stroke and TIA patients to obtain detailed information regarding the cognitive deficits. Future research projects should focus on the temporal dynamics of cognitive impairment after stroke and factors associated with cognitive impairment (including the role of strategic infarct locations), but also with cognitive recovery. This information might be important for patients and caregivers, as well as for the treating rehabilitation team.

## Supplemental Material

sj-docx-1-eso-10.1177_23969873221132032 – Supplemental material for Subacute cognitive impairment after first-ever transient ischemic attack or ischemic stroke in young adults: The ODYSSEY study Click here for additional data file. Supplemental material, sj-docx-1-eso-10.1177_23969873221132032 for Subacute cognitive impairment after first-ever transient ischemic attack or ischemic stroke in young adults: The ODYSSEY study by Mijntje MI Schellekens, Esther M Boot, Jamie I Verhoeven, Merel S Ekker, Mayte E van Alebeek, Paul JAM Brouwers, Renate M Arntz, Gert W van Dijk, Rob AR Gons, Inge WM van Uden, Tom den Heijer, Paul LM de Kort, Karlijn F de Laat, Anouk van Norden, Sarah E Vermeer, Marian SG van Zagten, Robert J van Oostenbrugge, Marieke JH Wermer, Paul J Nederkoorn, Frank G van Rooij, Ido R van den Wijngaard, Frank-Erik de Leeuw, Roy PC Kessels and Anil M Tuladhar in European Stroke Journal
