# Supplementary material for: Subacute cognitive impairment after first-ever transient ischemic attack or ischemic stroke in young adults: The ODYSSEY study
Source: Eur Stroke J. 2022 Oct 31;8(1):283–93. doi: 10.1177/23969873221132032 (PMC10069191; doi:10.1177/23969873221132032)

**Supplementary Table 1 Baseline characteristics of the study population en patients without cognitive assessment**

|  | **Patients with cognitive assessment (n=598)** | **Patients without cognitive assessment**  **(n=685)** | **P value** |
| --- | --- | --- | --- |
| Mean age at index event, years (SD) | 41.7 (7.7) | 41.8 (7.3) | 0.83 |
| Men, N (%) | 310 (51.8) | 364 (53.1) | 0.64 |
| Type index event, N (%) |  |  | 0.08 |
| TIA | 57 (9.5) | 47 (6.9) |  |
| Ischemic stroke | 541 (90.5) | 638 (93.1) |  |
| Median NIHSS score at admission (IQR) | 3 (1-5) | 3 (1-7) | <0,05 |
| Median NIHSS score at discharge (IQR) | 1 (0-2) | 1 (0-3) | <0,05 |
| TOAST, N (%) |  |  | 0.65 |
| Atherothrombotic | 25 (4.2) | 27 (3.9) |  |
| Likely atherothrombotic | 70 (11.7) | 96 (14.0) |  |
| Small vessel | 85 (14.2) | 81 (11.8) |  |
| Cardioembolic | 97 (16.2) | 124 (18.1) |  |
| Rare causes | 125 (20.9) | 149 (21.8) |  |
| Multiple causes | 37 (6.2) | 42 (6.1) |  |
| Cryptogenic | 159 (26.6) | 166 (24.2) |  |
| Vascular risk factors, N (%) |  |  |  |
| Hypertension | 224 (37.5) | 269 (39.3) | 0.51 |
| Diabetes mellitus | 55 (9.2) | 75 (10.9) | 0.30 |
| Dyslipidemia | 392 (65.6) | 458 (66.9) | 0.62 |
| Obesity | 78 (13.0) | 108 (15.8) | 0.17 |
| Morbid obesity | 32 (5.4) | 48 (7.0) | 0.22 |
| Smoking | 283 (47.3) | 356 (52.0) | 0.10 |
| Alcohol | 34 (5.7) | 54 (7.%) | 0.12 |

IQR: interquartile range. NIHSS: National Institutes of Health Stroke Scale; TOAST: Trial of ORG 10172 in Acute Stroke Treatment. Missing data in patients without cognitive assessment: NIHSS at admission 1 (0.1%); NIHSS at discharge 8 (1.2%).

**Supplementary Table 2 Reasons for non-completion for each test**

| **Cognitive test** | **Total non-completion** | **Technical problems** | **Physical disability** | **Severe cognitive impairment^a^** | **Refusal** |
| --- | --- | --- | --- | --- | --- |
| RAVLT trial 1-3 | 9 | 5 | 0 | 1 | 2 |
| RAVLT delayed recall | 19 | 11 | 0 | 2 | 6 |
| SDMT | 37 | 4 | 30 | 0 | 3 |
| Stroop part I | 26 | 14 | 6 | 4 | 2 |
| Stroop part II | 25 | 13 | 6 | 4 | 2 |
| ROCF copy | 31 | 1 | 28 | 1 | 1 |
| Verbal fluency | 13 | 6 | 0 | 3 | 4 |
| Stroop interference | 29 | 15 | 6 | 6 | 2 |
| Brixton test | 17 | 8 | 1 | 5 | 3 |
| Star Cancellation | 14 | 2 | 7 | 0 | 5 |
| Short token test | 30 | 16 | 2 | 4 | 8 |
| Digit span test | 28 | 9 | 0 | 4 | 15 |

Data were expressed as numbers. RAVLT: Rey Auditory Verbal Learning Test; SDMT: Symbol-Digit Modalities Test; ROCF: Rey-Osterrieth Complex Figure. a) Severe cognitive impairment that prevented the patient from understanding the instruction.

**Supplementary Table 3 Total number of cognitively impaired tests in a patient**

| **Total tests cognitively impaired** | **Total patients** |
| --- | --- |
| 0 | 132 |
| 1 | 145 |
| 2 | 116 |
| 3 | 67 |
| 4 | 52 |
| 5 | 30 |
| 6 | 23 |
| 7 | 13 |
| 8 | 13 |
| 9 | 2 |
| 10 | 3 |
| 11 | 2 |

**Supplementary Figure 1 Severity of subjective cognitive failures.**

**Supplementary Figure 2 Relation between subjective cognitively complaints score and number of cognitive impaired tests**


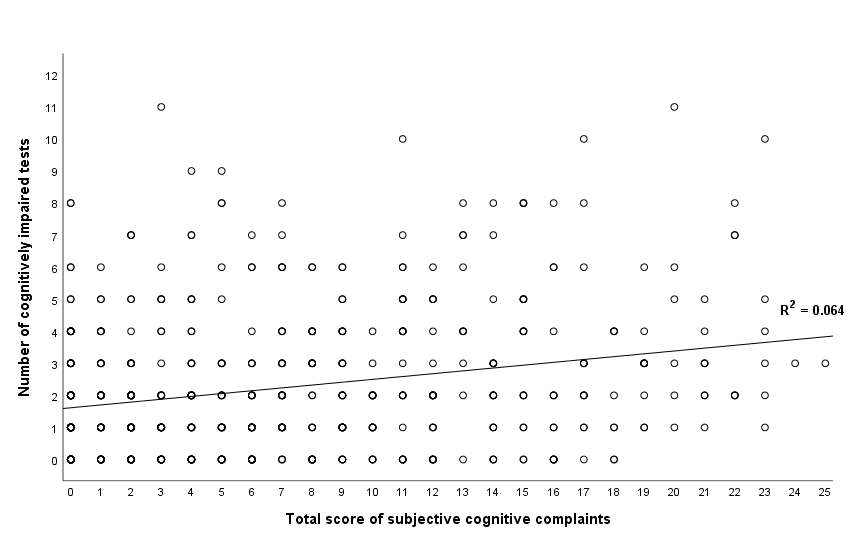

Supplement: sj-docx-1-eso-10.1177_23969873221132032 – Supplemental material for Subacute cognitive impairment after first-ever transient ischemic attack or ischemic stroke in young adults: The ODYSSEY study [file sj-docx-1-eso-10.1177_23969873221132032.docx]
